# Analysis of mutations in *EXT1* and *EXT2* in Brazilian patients with multiple osteochondromas

**DOI:** 10.1002/mgg3.382

**Published:** 2018-03-12

**Authors:** Savana C. L. Santos, Isabela M. P. O. Rizzo, Reinaldo I. Takata, Carlos E. Speck‐Martins, Jaime M. Brum, Claudio Sollaci

**Affiliations:** ^1^ Molecular Pathology Laboratory SARAH Network of Rehabilitation Hospitals Brasília Brazil; ^2^ Department of Clinical Genetics SARAH Network of Rehabilitation Hospitals Brasília Brazil; ^3^ Department of Orthopaedics SARAH Network of Rehabilitation Hospitals Brasília Brazil

**Keywords:** Brazilian population, chondrosarcoma, *EXT1*, *EXT2*, genotype, multiple osteochondromas

## Abstract

**Background:**

Multiple osteochondromas is a dysplasia characterized by growth of two or more osteochondromas. It is genetically heterogeneous, caused by pathogenic variants in *EXT1* or *EXT2* genes in 70%–90% of patients. The *EXT1* is more often mutated than *EXT2* gene, with a variable prevalence between populations. There are no data about *EXT1* and *EXT2* pathogenic variants in patients with multiple osteochondromas in Brazilian population. The aim of this survey is to characterize these to determine the genotype profile of this population.

**Methods:**

DNA sequencing (Sanger Method) and MLPA analysis were performed to identify point mutations and deletions/duplications in the sample of 153 patients in 114 families.

**Results:**

Germline variants were identified in 83% of families in which *EXT2* variants were detected in 46% and *EXT1* in 37% of cases. No variants were detected in 17% of them. We identified 50 different variants, 33 (13 frameshift, 11 nonsense, 5 missense, 2 splice site mutation, and 2 large deletions) in *EXT1* and 17 (6 frameshift, 6 splice site mutation, 3 nonsense, 1 missense, and 1 large deletion) in *EXT2*. Of all 50 variants, 31 (62%) were novel, including 20 out of 33 (60,6%) *EXT1* and 11 out of 17 (64.7%) *EXT2* alleles. The vast majority of variants (88%) were “loss‐of‐function” and two novel hotspots in *EXT2* gene were observed in our study.

**Conclusion:**

The prevalence of variants detected in the *EXT2* gene differs from other researches from Latin America, European, and Asian population. This uncommon prevalence could be related with the newly characterized variant hotspot sites detected in *EXT2* gene (p.Ala409Profs*26 and p.Ser290*). A high number of novel variants were also identified indicating that Brazilian population has a unique genetic profile. Characterizing this population and establishing its genotype is essential to understand the molecular pathogenesis of this disease in Brazil.

## 1 INTRODUCTION

Multiple osteochondromas (MO, OMIM 133700 and 133701) are a skeletal dysplasia characterized by development of two or more osteochondromas (cartilage‐capped bone tumors, always benign, which protrude from the metaphysis of long bone or flat bone surface; Hennekam, 1991). The median age of diagnosis is 3 years and it is based on clinical and radiological signs (Schmale, Conrad, & Raskind, 1994). Osteochondromas increase in number and size during growth and frequently cause short stature, bone deformity, pain, joint limitation, early osteoarthritis, peripheral nerve or tendon or blood vessel compression (Pedrini et al., 2011; Porter et al., 2004). The most serious complication is malignant transformation in chondrosarcoma, occurring in 1%–5% of cases (Pedrini et al., 2011; Porter et al., 2004; Schmale et al., 1994).

MO are genetically heterogeneous autosomal dominant disorder, with 100% of age‐related penetrance. The prevalence is estimated to be one in 50,000 North Americans, which is likely to be a conservative estimation due to a significant proportion of undiagnosed MO patients with no or mild unidentified symptoms (Schmale et al., 1994). MO are caused by pathogenic variants in two ubiquitously expressed tumor suppressor genes, *EXT1* (OMIM 608177, chr 8q24.11) and *EXT2* (OMIM 608210, chr 11p11.2) (Ahn et al., 1995; Stickens et al., 1996). The *EXT1* gene is composed of 11 exons and encodes for a protein of 746 amino acids (Ahn et al., 1995). The *EXT2* gene comprises 16 exons, but only exons 2 to 14 code for a product of 718 amino acids (Stickens et al., 1996). *EXT1* and *EXT2* are highly similar, specially in the carboxy terminal region that contains significantly less pathogenic variants (Ciavarella et al., 2013; Wuyts & Van Hul, 2000)). In the *EXT1* gene, missense mutations are common in or nearby regions that encode the highly conserved arginine residues at codons 280 and 340, which are located in the d‐glucuronic acid glycosyltransferase (d‐GlcA‐TII) catalytic domain of EXT1. Similarly, in *EXT2*, a recurrent pathogenic variant in exon 4 affecting an asparagine residue (p.Asp227Asn) is known and most pathogenic variants are found in the first eight exons (Heinritz et al., 2009; Philippe et al., 1997). EXT1 and EXT2 glycoproteins form a heterooligomeric complex that catalyzes the polymerization of the polysaccharide, heparan sulfate. This complex is an essential factor for multiple cellular functions, as growth signaling pathway, anchorage of extracellular matrix, signal transduction cascade for regulation of chondrocyte differentiation, ossification, and apoptosis (Hameetman et al., 2007; McCormick, Duncan, Goutsos, & Tufaro, 2000; Samuel, Costa, & Lindskog, 2014). The precise function of EXT1 and EXT2 in the development of MO has not been entirely understood (Jones, Pacifici, & Hilton, 2014; Samuel et al., 2014). Previous molecular analysis of the coding regions of both *EXT1* and *EXT2* genes described pathogenic variants in 46%–95% of affected individuals (Guo, Deng, & Liu, 2014; Jennes et al., 2009). The relative frequencies differed in *EXT1* and *EXT2* pathogenic variants among various ethnic groups. The frequency of pathogenic variants in *EXT1* was characterized to be 40%–75% versus 20%–40% in *EXT2* in Caucasian (French, British, German, Spanish, Belgian, Dutchman, Italian, Chilean, Argentine Caucasian) and Japanese populations (Delgado et al., 2014; Francannet et al., 2001; Heinritz et al., 2009; Ishimaru et al., 2016; Jennes et al., 2008; Lonie et al., 2006; Pedrini et al., 2011; Sarrión et al., 2013). In contrast, in Canadian and Chinese populations, the frequency of pathogenic variants in *EXT1* was 14%–30% versus 28%–50% in *EXT2* (Alvarez, Tredwell, De Vera, & Hayden, 2006; Guo et al., 2014; Xu et al., 1999). The 10 Canadian families that were studied by Alvarez had their ancestral origins in the UK (5), Germany (1), France (1), Japan (1), Austria (1), and India (1). Less than 20% of patients have a “de novo” mutation (Jennes et al., 2009; Schmale et al., 1994) and most of the identified pathogenic variants are inactivating mutations, including nonsense, frameshift, and splice site mutations (Jennes et al., 2009; Wuyts & Van Hul, 2000).

To date, the profile of mutations in *EXT1* and *EXT2* genes in Brazilian patients with MO is unknown. The objective of this survey is to identify, describe, and analyze variants in *EXT1* and *EXT2* genes of 153 Brazilian patients with MO recruited at our rehabilitation center.

## 2 MATERIALS AND METHODS

### 2.1 Ethical compliance

This study was approved by the National Committee of Ethics in Research, SARAH Network of Rehabilitation Hospitals. All the patients or their parents (if the patient was underage) signed informed consent and approved the anonymous use of clinical and molecular data for the present diagnostic study.

### 2.2 Patients and clinical data

We identified 153 patients in 114 families with a clinical diagnosis of MO (89 unrelated and 25 families) attended at the SARAH Network of Rehabilitation Hospitals in Brasília from 2012 to 2014.

The inclusion criteria for this study were to be affected by MO, to be SARAH's patient, and to consent to the research. A retrospective study of clinical, surgical, and radiological data of these patients was made. The diagnosis of MO was established when radiologically at least two osteochondromas of the juxta‐epiphyseal region of long bones were observed. The diagnostic of malignant transformation was obtained by radiological investigation and histopathological examination.

Brazilians form one of the most heterogeneous populations in the world, the result of 5 centuries of interethnic crosses between peoples from three continents: the European immigration, represented mainly by the Portuguese; African slaves; and the autochthonous Amerindians (Alves‐Silva et al., 2000). The total sample showed nearly equal amounts of Native American, African, and European matrilineal genetic contribution but with regional differences (Alves‐Silva et al., 2000). This study included patients from all regions of the country, reducing ethnic differences. The age of patients ranged from 3 to 75 years old and the median age was 20. The prevalence was higher in males (ratio = 1.22/1) and familial cases (63%). Seven unrelated patients aged between 21 and 54 presented malignant degeneration to chondrosarcoma. Histological analyses were made in six of them: four had grade I and two had grade II according to World Health Organization criteria.

### 2.3 Genotyping and mutation analysis

Genomic DNA was extracted from peripheral blood samples according to a standard protocol (Miller, Dykes, & Polesky, 1988). The codifying and flanking regions of all exons of *EXT1* and *EXT2* genes were amplified by PCR. Primers were designed by Primer Blast Designing Tool (http://www.ncbi.nlm.nih.gov/tools/primer-blast) and are listed in Table S1. Exon 1 of *EXT1* and exon 2 and 10 of *EXT2* were split into several overlapping fragments in order to obtain amplification products that did not exceed 300 bp. PCR was performed in a 50 μl reaction volume, containing 50 ng of DNA genomic, 1× PCR buffer, 1.5–2 mmol/L MgCl_2_, 0.2 mmol/L of each dNTP, 0.25 μmol/L of each forward and reverse primer, and 0.5 U of platinum Taq polymerase (Invitrogen, Carlsbad, CA). All PCR programs included an initial denaturation of 5 min at 95°C, followed by 35 cycles of 30 s at 95°C, 30 s at annealing temperature, and 1 min at 72°C. The annealing temperature was 62°C for all primer combinations. We first analyzed 153 patients in 114 families for *EXT1* variants by denaturing high‐performance liquid chromatography (dHPLC) using WAVE DNA Fragment Analysis System (Transgenomic, Crewe, UK). Samples with abnormal elution profile were directly sequenced (Big Dye Terminator Cycle Sequence kit v.3.0 and ABI3130xl automated Sequencer, Applied Biosystems) in both directions with forward and reverse primers on the original amplicon and on different PCR product. Negative cases were then screened for *EXT2* variants using the same approach. Negative cases for both genes were directly sequenced. If no mutation was detected, negative cases were analyzed by MLPA (Multiplex ligation‐dependent probe amplification—SALSA MLPA kit P215‐B1 EXT, MCR‐Holland, Amsterdam, The Netherlands). Variants were searched in the database Osteochondromas Mutation Database (MOdb) (http://medgen.ua.ac.be/LOVD) (last accessed on October 2016).

The reference *EXT1* and *EXT2* sequences were obtained from GenBank with accession numbers NM_000127.2, NP_000118.2, NM_001178083.1, NP_001171554.1, respectively. Mutation numbering is based on cDNA CCDS6324.1 and CCDS53618.1, respectively.

The pathogenic role of novel missense mutations that were neither found in MOdb nor ExAC or 1,000 Genomes (https://www.ncbi.nlm.nih.gov/variation/tools/reporter) was evaluated by testing other family members, when available, and by three in silico online tools: PolyPhen‐2 (Polymorphism Phenotyping v2; http://genetics.bwh.harvard.edu/pph2/, last accessed on October 2016), MutPred (v.1.2; http://mutpred.mutdb.org/, last accessed on October 2016), and Mutation Taster2 (http://www.mutationtaster.org, last accessed on October 2016). The Human Splice Finder (http://www.umd.be/HSF/, last accessed October 2016) online tool was used to assess the possible effect of novel intronic variants on splicing sites.

The nomenclature of novel variants was given according to the official HGVS guideline (http://www.hgvs.org) and based on the GenBank accession number.

## 3 RESULTS

Exons and flanking regions of the *EXT1* and *EXT2* genes were analyzed from the genomic DNA of 153 patients in 114 families with MO and MLPA analyses were performed in DNA samples with negative results for sequencing analysis. A mutant allele was found in 83% (95/114) of unrelated MO patients in which *EXT2* variants were detected in 46% (53/114) and *EXT1* variants were detected in 37% (42/114) of cases. We identified 50 different variants: 33 (13 frameshift, 11 nonsense, 5 missense, 2 splice site mutation, and 2 large deletions) in *EXT1* and 17 (6 frameshift, 6 splice site mutation, 3 nonsense, 1 missense, and 1 large deletion) in *EXT2* gene. Of all 50 variants, 31 (62%) were novel with no description in the Multiple Osteochondroma Mutation Database (MOdb) (http://medgen.ua.ac.be/LOVDDv.2.0/home.php). Twenty out of 33 (60.6%) *EXT1* variants were novel as were 11 of 17 (64.7%) of *EXT2* variants (Table 1).

**Table 1:** (a) List of mutations in *EXT1* in MO patients (HPO id 0002762). (b) List of mutations in *EXT2* in MO patients (HPO id 0002762)

Family number^⊤^	Exon–intron	DNA	Deduced protein change	Mutation type	Publication
(a)					
P01	Exon 1	c.9insA	p.Lys6Thrfs*29	Frameshift	This study
P02	Exon 1	c.125delG	p.Gly42Valfs*93	Frameshift	This study
P03	Exon 1	c.173_174insG	p.Phe58Leufs*72	Frameshift	This study
		c.186_192delCGCCC			
P05,P06, P07	Exon 1	c.651_672del22	p.Lys218Alafs*27	Frameshift	This study
P08	Exon 1	c.859delC	p.His287Ilefs*	Frameshift	This study
P10	Exon 1	c.774_775delCT	p.Pro258Profs*29	Frameshift	This study
P20, P21, F8, F24	Exon 6	c.1469delT	p.Leu490Argfs*9	Frameshift	Signori et al. (2007)
P22, P23	Exon 6	c.1468_1469insC	p.Leu490Profs*31	Frameshift	Signori et al. (2007)
P25	Exon 8	c.1642delA	p.Ser548Alafs*73	Frameshift	Lonie et al. (2006)
F21	Exon 8	c.1680_1682insGC	p.Val561Profs*16	Frameshift	This study
P26	Exon 8	c.1718_1719delCA	p.Thr573Argfs*53	Frameshift	This study
F14	Exon 10	c.2040_2041delGA	p.Thr681Aspfs*?	Frameshift	This study
P27	Exon 10	c.2001delG	p.Leu667Phefs*5	Frameshift	This study
F22	Exon 10	c.1896_1898delCCT	p.Tyr632*	Nonsense	This study
F12	Exon 1	c.289A>T	p.Lys97*	Nonsense	This study
P04	Exon 1	c.385G>T	p.Glu129*	Nonsense	This study
F15, P16	Exon 4	c.1236G>A	p.Trp412*	Nonsense	This study
P18	Exon 5	c.1320insT	p.Asp441*	Nonsense	Gigante et al. (2001)
P19	Exon 5	c.1335G>A	p.Trp445*	Nonsense	This study
F17	Exon 6	c.1522C>T	p.Gln508*	Nonsense	Pedrini, not published
F7	Exon 7	c.1550G>A	p.Trp517*	Nonsense	Jennes, not published
P24	Exon 8	c.1696G>T	p.Glu566*	Nonsense	Vink et al. (2005)
F23	Exon 10	c.1911C>G	p.Tyr637*	Nonsense	This study
P29	Exon 10	c.1902C>G	p.Tyr634*	Nonsense	This study
P14	Intron 2	c.1056+1G>A		Splice Site	Jennes et al. (2008)
P28	Intron 9	c.1884‐2A>G		Splice Site	This study
F04	Exon 1	c.803G>A	p.Gly268Glu	Missense	This study
P09	Exon 1	c.791T>G	p.Leu264Arg	Missense	This study
P11, P12, P13	Exon 2	c.1019G>T	p.Arg340Leu	Missense	Jennes et al. (2008)
F9	Exon 2	c.1019G>A	p.Arg340His	Missense	Pedrini et al. (2005)
P15	Exon 2	c.1037G>C	p.Arg346Thr	Missense	Delgado et al. (2014)
P70		Del EXT1		Deletion	Hall et al. (2002)
P71	Exon 1	ex1del		Deletion	Jennes, not published
(b)					
P34	Exon 2	c.202delG	p.Val68Leufs*43	Frameshift	This study
P35	Exon 2	c.453insT	p.Gly152Trpfs*13	Frameshift	This study
P37	Exon 3	c.539_540insTC	p.Asp181Glyfs*89	Frameshift	This study
P38	Exon 3	c.550_551dupGT	p.Thr184Valfs*86	Frameshift	This study
P51	Exon 5	c.760delC	p.Leu255Cysfs*15	Frameshift	This study
F1, F2, F5, F18, F19, F20, F25, P56, P57, P58, P59, P17, P60, P61, P62, P63, P64, P65, P66	Exon 8	c.1225delG	p.Ala409Profs*26	Frameshift	This study
F10, F13, P30, P31, P32, P33,	Exon 2	c.151G>T	p.Glu51*	Nonsense	Lonie et al. (2006)
F11, F16, P41, P42, P43, P44, P45, P46, P47, P48, P49	Exon 5	c.869C>A	p.Ser290*	Nonsense	This study
F6, P67, P69	Exon 8	c.1182G>A	p.Trp394*	Nonsense	Delgado et al. (2014)
P36	Intron 3	c.626+1G>A		Splice Site	Vink et al. (2005)
P39	Intron 3	c.626+2delT		Splice Site	This study
P40	Intron 4	c.743+1G>A		Splice Site	Vink et al. (2005)
P50	Intron 4	c.744‐13del14insATTC		Splice Site	This study
P52, P53	Intron 6	c.1079+1G>A		Splice Site	Jennes, not published
P54	Intron 6	c.1173+1dupG		Splice Site	Francannet et al. (2001)
P55	Exon 7	c.1110G>T	p.Met370Ile	Missense	This study
F26	Exon 6‐8	ex6_ex8_del		Deletion	This study

⊤: P means individual case and F, familial nucleus.

All identified variants were submitted to ClinVar with ACMG 2015 classification criteria (Richards et al., 2015).

Of the 50 different variants identified, 39 (78%) were found in only one individual. Recurrent pathogenic variants observed in patients of Brazil included two novel hotspots in *EXT2* gene. The most frequent pathogenic variant detected in *EXT1* gene was p.Leu490Argfs*9 occurring in four families, followed by p.Arg340Leu and p.Lys218Alafs*27 both occurring in three families. Only, p.Lys218Alafs*27 was novel. Two novel hotspots in *EXT2* gene were observed in our study: p.Ala409Profs*26 detected in 19 families, and p.Ser290Ter detected in 11 families (Table 1).

The vast majority of variants (88%) were “loss‐of‐function” mutation (frameshift, nonsense, splicesite, and large deletions) responsible for the production of truncated protein products. Two out of five missense mutations detected in *EXT1* gene were novel. Bioinformatic predictions suggested a pathogenic role for these alterations. In particular, p.Leu264Arg change from a nonpolar amino acid (Leu) to a basic amino acid (Arg) was considered “probably damaging” by Polyphen2 (score of 0.997; sensitivity: 0.27; specificity: 0.98), “deleterious mutation” by MutPred (score: 0.851, with a confident hypothesis of a gain of methylation at Leu264Arg; *p* = .0089), and “disease causing” by the Mutation Taster (score: 0.999, amino acid sequence changed, protein features (might be) affected, splicesite changes). Also, this variant was neither found in ExAC nor 1,000 Genomes (https://www.ncbi.nlm.nih.gov/variation/tools/reporter); a variant at same codon (p.Leu264Pro) was previously described (Delgado et al., 2014). Similarly, the missense mutation, p.Gly268Glu, changed from a nonpolar amino acid (Gly) to a polar amino acid (Glu) was predicted as “possibly damaging” by Polyphen2 (score: 0.856; sensitivity: 0.72; specificity: 0.88). In addition, it was predicted as “deleterious mutation” by MutPred (score: 0.927, with a confident hypothesis of a loss of a MoRF binding; *p* = .0188) and as “disease causing” by the Mutation Taster (score: 0.999, amino acid sequence changed, protein features (might be) affected, splice site changes). Also, it was submitted as likely pathogenic at ClinVar (https://preview.ncbi.nlm.nih.gov/clinvar/variation/265126/). Only one novel missense mutation was detected in *EXT2* gene. There is no amino acid classification change in the p.Met370Ile mutation detected where both Methionine (Met) and Isoleucine (Ile) are nonpolar amino acids. As expected, bioinformatics predictions suggested a benign role for this variant in all three tools tested (Polyphen2, MutPred, and Mutation Taster) as well was described as likely benign according to ClinVar criteria (https://www.ncbi.nlm.nih.gov/clinvar/variation/134217/).

Only one novel intronic variant in *EXT1* gene was detected. In silico analyses of c.1884‐2A>G predicted the use of cryptic acceptor splice sites by Human Splice Finder (score: −83.68%) located between 11 nucleotides upstream and three nucleotides downstream from the wild‐type sequence with an alteration of the WT acceptor site, most likely affecting splicing. On the other hand, two novel intronic variants were found in *EXT2* gene. The Human Splice Finder analysis of c.626+2delT variant predicted the use of cryptic donor splice site (score: −157.2%) located 88 nucleotides downstream from the wild‐type sequence with an alteration of the WT donor site, possibly affecting splicing. The other intronic variant in *EXT2* gene, c.744‐13del14insATTC, was predicted by Human Splice Finder as an alteration of the WT acceptor site, most probably affecting splicing located (score −50.05%) 12 nucleotides upstream from the wild‐type sequence.

**Figure 1:**
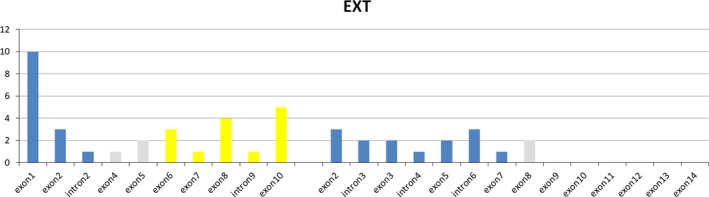
The position and frequency of the pathogenic variants in *EXT1* and *EXT2* genes

The position of the pathogenic variants in *EXT1* gene showed that the same number of *EXT1* pathogenic variants were located in regions that encode the exostosin domain (45.2%—from amino acid 111 to 396) and the glycosyltransferase domain (45.2%—from amino acid 480 to 729) of the EXT1 protein (Figure 1). In contrast, 87.5% (14/16) of *EXT2* variants were located in regions that encode the exostosin domain (from exon 2 to 7) and only 12.5% (2/16) were located in regions that encode the middle segment of the *EXT2* gene, both specifically in the exon 8, considering that no variants were located in glycosyltransferase domain (from exon 10 to 14, Figure 1). Deletions in *EXT1* or *EXT2* genes were not considered in these frequencies.

Large deletions were detected by the MLPA technique in 3 of 114 (2.6%) families. In the *EXT1* gene, we found one patient with a deletion of exon 1 and the other with a complete deletion of the gene. The deletion of 3 exons in the *EXT2* gene (from exon 6 to exon 8) was detected in one family. No mutation was found in *EXT1* or neither *EXT2* gene in 19 (17%) families.

Seven individuals unrelated of 114 families (6%) presented malignant transformation to chondrosarcoma (Table 2). The degree of anaplasia was not determined in F24 family, who presented p.Leu490Argfs*9 variant in *EXT1* gene. Chondrosarcoma grade I was diagnosed in four families. The P03 and F14 families bore novel and no recurrent frameshift mutations (p.Phe58Leufs*7 and p.Thr681Aspfs*, respectively) in *EXT1* gene; P74 and P75 families had no *EXT1/EXT2* variants identified. Malignant transformation to chondrosarcoma grade II was detected in F19 and F6 families, who presented pathogenic variants in *EXT2* gene (p.Ala409Profs*26 and p.Trp394*, respectively).

**Table 2:** Characteristics of patients with chondrosarcoma

Gender	Family number	Gene	Age at chondrosarcoma diagnosis	Location	Mutation type	Exon	DNA	Deduced protein change	Publication	Histopathologic grading of anaplasia
Female	F14	*EXT1*	29	Humerus	Frameshift	10	c.2040_2041delGA	p.T681DfsX	This study	Grade I chondrosarcoma
Male	F19	*EXT2*	28	Iliac	Frameshift	8	c.1225delG	p.A409PfsX26	This study	Grade II chondrosarcoma
Female	—	Non‐ *EXT1*/Non‐ *EXT2*	22	Femur						Grade I chondrosarcoma
Female	—	*EXT1*	54	Humerus	Frameshift	1	c.173_174insG, c.186_192delCGCCC	p.F58LfsX72	This study	Grade I chondrosarcoma
Female	F6	*EXT2*	30	Iliac	Nonsense	8	c.1182G>A	p.W384X	Delgado et al. (2014)	Grade II chondrosarcoma
Male	F24	*EXT1*	40	Tibia	Frameshift	6	c.1469delT	p.L490RfsX9	Signori et al. (2007)	
Female	—	Non‐ *EXT1*/Non‐ *EXT2*	21	Iliac						Grade I chondrosarcoma

## 4 DISCUSSION

**Figure 2:**
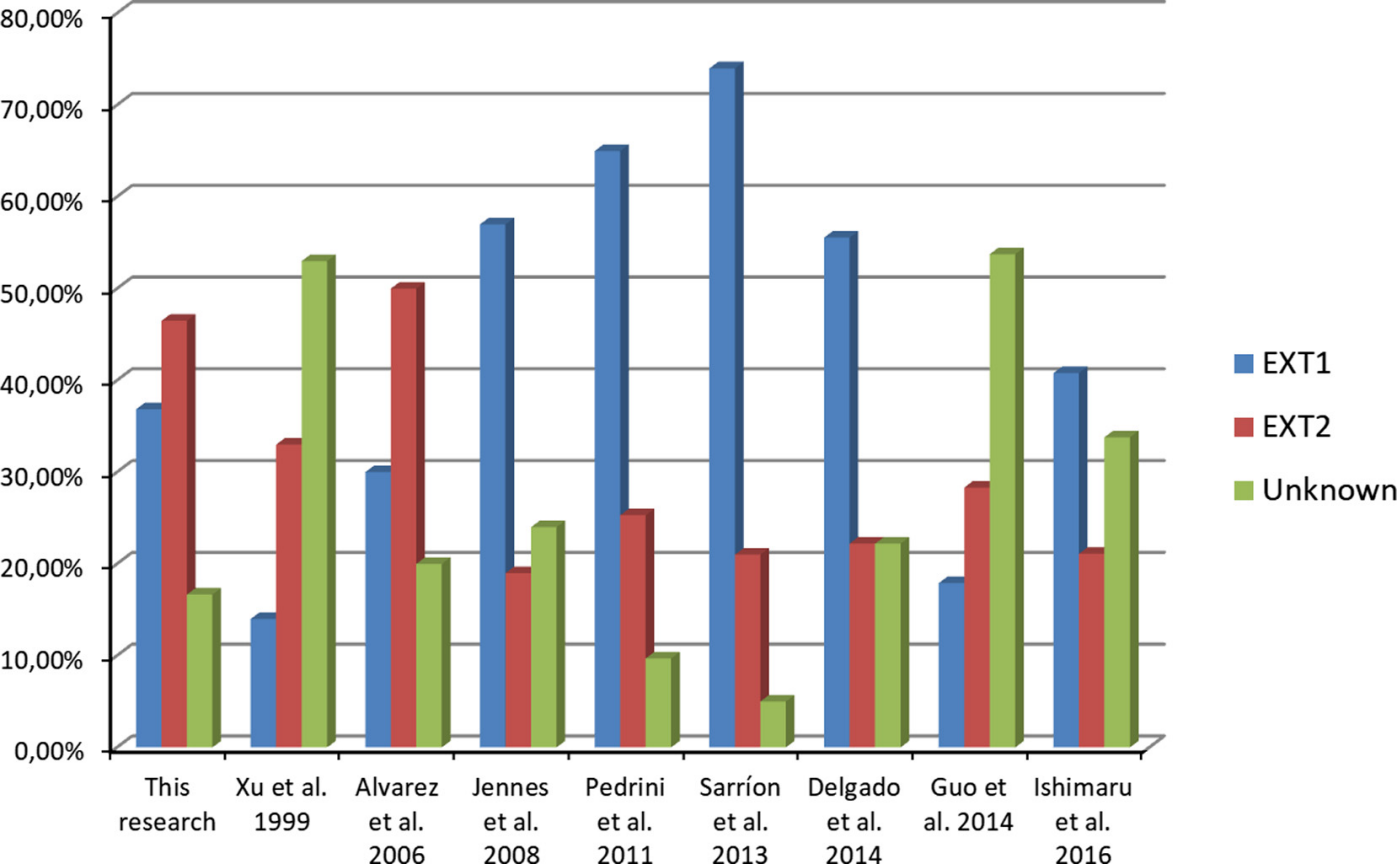
The frequency of pathogenic variants in *EXT1* and *EXT2* genes, and unknown cause observed in this research and other populations

This study is the first Brazilian research in MO, with a broad spectrum of variants detected in *EXT1* and *EXT2* genes affected. Variants were found in 83% of MO families. *EXT2* variants were detected in 46% (53/114) and *EXT1* variants were detected in 37% (42/114) (Figure 2). These results differ from those observed in other populations, in which *EXT1* gene was responsible for the vast majority of MO cases (Delgado et al., 2014; Jennes et al., 2008; Pedrini et al., 2011; Sarrión et al., 2013). So far, the evaluation of *EXT1* and *EXT2* genes in Canadian and Chinese populations with MO were the only reports that identified a higher frequency of mutations in *EXT2* than *EXT1* gene (Alvarez et al., 2006; Guo et al., 2014; Jennes et al., 2009; Xu et al., 1999). Canadian group considered that one of the main limitations of their study was the small sample size (Alvarez et al., 2006). Although Xu et al. (1999) and Guo et al. (2014) attributed the differences in involvement of *EXT1* and *EXT2* genes in Chinese population to the genomic variance, the scope of gene segments tested, the inconsistency in variant screening methods, and the presence of hotspots in *EXT1* was observed only in Caucasian and Japanese patients.

In contrast to a Latin America study recently published, which evaluated Chileans and Argentines whose origins are basically European and indigenous (Delgado et al., 2014), we observed recurrent pathogenic variants in patients of Brazil. The two most frequent pathogenic variants in *EXT2* gene have not been previously reported. Both of them may be considered new hotspots in *EXT2*, which may contribute for the fact that *EXT2* mutations are more frequent in Brazilian patients. The frameshift mutation p.Ala409Profs*26 was detected in 19 families and the nonsense mutation p.Ser290Ter was detected in 11 families with *EXT2* mutated allele.

On the other hand, the two most frequent pathogenic variants detected in *EXT1* gene (p.Leu490Argfs*9 and p.Arg340Leu) have previously been reported in European populations and both of them were already mentioned to be located in mutation hotspots (Ciavarella et al., 2013; Francannet et al., 2001; Jennes et al., 2008; Philippe et al., 1997). The Brazilian population is a mixed population that consists of a high influence of Europeans in its colonization, which could be responsible for the presence of recurrent alterations in hotspots found in our study. Unfortunately, there are no similar researches about Amerindians or African population to compare with our results.

As previously reported in distinct populations (Ciavarella et al., 2013; Delgado et al.,^2014^; Heinritz et al., 2009; Ishimaru et al., 2016; Pedrini et al., 2005; Sarrión et al., 2013) a high number of novel pathogenic variants was also identified in Brazilian patients. Of all 50 different variants found in this study, 31 (62%) were novel with no description in the Multiple Osteochondroma Mutation Database (MOdb) (http://medgen.ua.ac.be/LOVDDv.2.0/home.php). Twenty out of 33 (60,6%) *EXT1* variants were novel as were 11 of 17 (64.7%) of *EXT2* variants (Table 1). This fact confirms the strong allelic heterogeneity of *EXT1/2* genes in MO patients. Moreover, inactivating mutations (frameshift, splice, and nonsense mutations and large deletions), which results in the formation of nonfunctional EXT1 and EXT2 proteins and represent the most common MO‐causing variants, corresponded to 88% of variants found in this study concurring with other reports previously published (Ciavarella et al., 2013; Delgado et al., 2014; Guo et al., 2014; Jennes et al., 2008, 2009; Pedrini et al., 2005, 2011; Sarrión et al., 2013; Wuyts & Van Hul, 2000) (Table 1). Two out of five exonic missense mutations detected in *EXT1* gene (p.Leu264Arg and p.Gly268Glu) and the only one exonic missense mutation detected in *EXT2* gene (p.Met370Ile) were novel. Both variants in *EXT1* are predicted to be deleterious, while the variant found in *EXT2* was considered benign according to three different in silico analysis tools. Splice site variants were detected in two patients in *EXT1* gene. One of them, c.1884‐2A>G, was a novel variant observed in one unrelated patient. Use of cryptic acceptor splice sites was predicted by in silico analysis. On the other hand, seven splice site variants were identified in *EXT2* gene. Two of them were novels: c.626+2delT and c.744‐13del14insATTC, both in one unrelated patients (Table 1). In silico analysis predicted the use of alternative cryptic donor splice site to both variants, c.626+2delT and c.744‐13del14insATTC.

All missense mutations were present in exon 1 and 2 of *EXT1* gene. Both of them are located in exons that encode the exostosin domain of EXT1 protein (from exon 1 to exon 3) suggesting that the amino acids involved are crucial for proper functioning of it.

Analysis of the distribution of variants over the *EXT1* gene reveals that alterations in Brazilian patients are equally distributed over the exons 1 to 3 which encode aminoacids of exostosin domain (45.2%) and over the exons 6 to 10 which encode aminoacids of glycosyltransferase domain (45.2%) of EXT1 protein. The remaining variants were found in exons 4 and 5 (9.6%) which encode aminoacids of the middle segment of EXT1 protein. This pattern of distribution was not observed in studies previously reported where most of variants are located in exons 1 to 3 (Ciavarella et al., 2013; Delgado et al., 2014; Francannet et al., 2001; Signori et al., 2007; Wuyts & Van Hul, 2000; Xu et al., 1999). These data suggest that variants that modify the glycosyltransferase domain may be as relevant as variants that modify exostosin one despite the COOH‐terminal catalytic domains. In contrast, the vast majority of *EXT2* variants (87.5%) found in this report are located in regions that are involved in encoding aminoacids of exostosin domain (exons 2 to part of 7), and no variation was found in regions that are involved in encoding aminoacids of glycosyltransferase domain (part of exon 9–14). Similar observations have been reported in different European and Asian populations analyzed by several authors (Ciavarella et al., 2013; Francannet et al., 2001; Pedrini et al., 2005; Sarrión et al., 2013; Signori et al., 2007; Wuyts & Van Hul, 2000; Xu et al.,^1999^) but not in a Latin American study in which *EXT2* pathogenic variants were more frequent in the last exons (Delgado et al., 2014). A genotype–phenotype evaluation could provide a better understanding of the relevance and the role of exostosin and glycosyltransferase domains in MO patients.

MLPA analysis was able to detect three (2.6%) families with large deletions, two in *EXT1* gene and one in *EXT2* gene. Exon 1 has been previously reported as a hotspot in MO patients where a exon1 deletion has been found (Delgado et al., 2014; Signori et al., 2007; Vink et al., 2005) and a deletion of the entire *EXT1* gene was previously reported as well (Hall, Cole, Haynes, & Hecht, 2002; Jennes‐not published). However, further analysis needs to be carried out to determine whether the breakpoints in all cases are the same. A novel deletion of three exons in *EXT2* gene (from exon 6 to exon 8) was found in one family. These findings show a slightly lower frequency of large deletions found in other studies where 4% to 9% of deletions in one or more exons are observed (Delgado et al., 2014; Jennes et al., 2008; Pedrini et al., 2011; Signori et al., 2007; White et al., 2004).

No variant was found in *EXT1* or *EXT2* genes in 17% of families after a multistep variant screening was performed. The results are consistent with other studies reporting that 10%–30% of cases present no alterations in both *EXT1* and *EXT2* (Boveé, 2008; Ciavarella et al., 2013; Gigante et al., 2001; Jennes et al., 2008; Lonie et al., 2006; Pedrini et al., 2005, 2011). There are some plausible reasons for the lack of *EXT* gene mutations. First, genetic heterogeneity must be considered, and the involvement of other still unidentified genes cannot be ruled out (Ciavarella et al., 2013; Ishimaru et al., 2016; Jennes et al., 2012; Sarrión et al., 2013; Wuyts & Van Hul, 2000). Second, we have not evaluated the presence of variants in the promoter region, the 5′untranslated region (5′UTR) nor the 3′UTR of both genes. Previous studies have shown that variant analysis of the open reading frame of *EXT1* did not detect pathogenic variants in several families known to be linked to the EXT1 locus (Hecht et al., 1997; Wells et al., 1997) which suggests the existence of some pathogenic variants outside the EXT1 coding region. Moreover, the description of a regulatory role of SNP (rs34016643) within transcription factor binding site of *EXT1* gene that results in a relevant increase in EXT1 promoter activity was reported (Jennes et al., 2012). Furthermore, the presence of EXT mosaic pathogenic variants in a fraction of the patients must be considered as a putative cause of undetected EXT mutations in MO cases (Sarrión et al., 2013; Szuhai et al., 2011). Finally, intronic deletion and duplication in *EXT1* detected only by array‐CGH was revealed as a new causative mechanism for MO (Waaijer et al., 2013). According to this, screening with array‐CGH and next‐generation sequencing to study additional causative mechanism should be performed.

The most serious complication in MO is malignant transformation to a chondrosarcoma, occurring in 1%–5% of cases. It is interesting to note that, in our cohort, malignant progression occurred in 6% of families affecting both sporadic cases and family nuclei which is not in agreement with other authors (Pedrini et al., 2011; Porter et al., 2004; Schmale et al., 1994). Irrespective of *EXT1* pathogenic variant or no *EXT1/EXT2* pathogenic variant identified, the histopathology classification of anaplasia of their tumors was grade I. The two patients who presented loss‐of‐function mutations in exon 8 of *EXT2* gene underwent malignant degeneration to chondrosarcoma grade II, which may suggest that mutations in *EXT2* gene may be responsible for a propensity of MO patients to develop tumors that are more aggressive. Our data did not agree with other studies in which an increased rate of malignant transformation in patients with *EXT1* pathogenic variants was observed (Alvarez et al., 2006; Porter et al., 2004).

In conclusion, the genotype characterization of a wide number of MO patients suggests that Brazilian population has a unique genetic profile that differs from the European and Latin American populations. The identification of a high number of novel pathogenic variants, presence of a higher frequency of *EXT2* than *EXT1* pathogenic variants, detection of two novel hotspots in *EXT2* gene, and the different pattern of distribution of pathogenic variants over the EXT1 and EXT2 protein indicate the uniqueness of our population.

Further research to detect mosaic alterations in mutation‐negative patients, to study the genotype–phenotype correlation of these patients and to perform the genotyping of chondrosarcoma will contribute to elucidate this complex disease.

## CONFLICT OF INTEREST

The authors declare that there is no conflict of interest with any financial organization or corporation or individual that can inappropriately influence this work.
