# Supplementary material for: Analysis of mutations in EXT1 and EXT2 in Brazilian patients with multiple osteochondromas
Source: Mol Genet Genomic Med. 2018 Mar 12;6(3):382–92. doi: 10.1002/mgg3.382 (PMC6014457; doi:10.1002/mgg3.382)
Supplement: Supplementary file 1 [file MGG3-6-382-s001.docx]

| **Online Supplementary Table S1. Primers used to amplify the exons of the *EXT1* and *EXT2* genes** | | | | | |
| --- | --- | --- | --- | --- | --- |
| Gene | Exon | Upstream primer 5'-3' | Downstream primer 5'-3' | Amplicon length (bp) |  |
| EXT1 | Exon 1_1 | CAATCCTCTTGACCCAGGC | AAATGATCCGGACTGGGGTG | 187 |  |
|  | Exon 1_2 | CACCCCAGTCCGGATCATTT | TCTCCCCTTTTTGCTGTGGG | 235 |  |
|  | Exon 1_3 | CCCACAGCAAAAAGGGGAGA | TGGAGACTCTGCACTTTGGA | 189 |  |
|  | Exon 1_4 | AGACCAGTTGTCACCTCAGT | CCCTCCTGTCCTGGGATGAT | 250 |  |
|  | Exon 1_5 | AGGGGAGAGGGGGTTTTTGA | GCCCAGACACTTACTTCTCATACT | 242 |  |
|  | Exon 2 | CCCACATTCGCAATGAGTCTTC | TCCTCAGCCCTATTCTGGGA | 185 |  |
|  | Exon 3 | TCTGCTGTCGCTTTCCTCAC | GACGGGGGCAGCAATAATCT | 209 |  |
|  | Exon 4 | ATTCCTATGTGTTCACCTTGTGT | TCAAGCCCAAGAGCCAAGT | 179 |  |
|  | Exon 5 | TCTCATCCTCACTGTGTGCT | AGGGTAAACAAGGGCAACTCC | 209 |  |
|  | Exon 6 | CTTGCTTTCCAGCGCTTCAT | GTAAGGAGGGCGGAGTCTCT | 235 |  |
|  | Exon 7 | ACATCCTACCCCAGCCATCT | CCCATGGAGAAACCAAGGCT | 196 |  |
|  | Exon 8 | CCTCCCCACTGCCTACTTCTA | AACATGAGGTGACTGCCTGA | 179 |  |
|  | Exon 9 | GTTTGGCTTGTGTTCTGCCT | TAAAGTCTGTAAGAGACATGTCCAG | 219 |  |
|  | Exon 10 | ATGTGATAATGGCCCCCTGTG | GTGAAGCAAGGGAAGAGGGC | 250 |  |
|  | Exon 11 | TCCATCTCACCTTGCACTTCT | CTTCCCCTCCCCCACTCAG | 270 |  |
| EXT2 | Exon 2_1 | TTCAAGTGTCATTTGCCATCCT | GGACAATGGAGAAGAGGGTGA | 191 |  |
|  | Exon 2_2 | CTTCTCCATTGTCCTCCTGGG | TTCTGCAACTGAGATCCCCC | 173 |  |
|  | Exon 2_3 | CGTGATGTGCCGGTTGTTAG | TTGCTGACAGAGACGCCAAA | 182 |  |
|  | Exon 2_4 | GGATGACTTTGGCGTCTCT | GGGCCACTCAAGTATCTCCT | 241 |  |
|  | Exon 3 | GGCTTGGGGATCCTTGATAGTT | CCCATCATAAGGACAGCCCC | 220 |  |
|  | Exon 4 | CCTCTCCACAGTGTGTATCAGA | ACACCTGGCTGGGATGAATG | 214 |  |
|  | Exon 5 | ACCAGCTGCAATTTTCCAATCA | AACTCTCCTGAGCCTTTGCG | 280 |  |
|  | Exon 6 | TTTGTAATCTCTTGCCTCTTTGTGT | ATACGCAGAACCACTAATGTAGA | 219 |  |
|  | Exon 7 | ATGCTTTCTGTGAAGGGCTGT | TCCAGTCAAGGCCACCATTT | 208 |  |
|  | Exon 8 | TTTCCCACTCTGTCTCGCTTG | TTTTCCTTCCACCCACCCTGA | 249 |  |
|  | Exon 9 | ACAGCTGCTTTTCTGACCCG | CCAGCTGAGAGAGGCACTAA | 261 |  |
|  | Exon 10_1 | CCATCTCATTTGTGATGTCATGCT | ATTGCAGCTCGTCAGAGGTC | 242 |  |
|  | Exon 10_2 | TCTCTCTGGCCCAAAATCCG | AAGACAAGCAGTCATAGGAAGT | 227 |  |
|  | Exon 11 | CAGCACTGAATGGTTGCTGTC | TTTTGTCACCTTGCCAGGACT | 237 |  |
|  | Exon 12_1 | CTTGGCTATGCTGCCCCTTA | CTGCTTTTCCCGTGACGTTG | 201 |  |
|  | Exon 12_2 | TCAAGAACTGGGTAGATGCTCA | TTTCCCAATGTGACCGCATC | 248 |  |
|  | Exon 13 | AGTGTGGTGTCACAAGCATGA | TAAGGCGCACTTTTGGTTGG | 189 |  |
|  | Exon 14 | ATCTTTTCTCCCTGCCCCCAT | TCTGTCCCAGCCTCACATTCA | 255 |  |
